# Studies on a Novel Serine Protease of a Δ*hapA*Δ*prtV Vibrio cholerae* O1 Strain and Its Role in Hemorrhagic Response in the Rabbit Ileal Loop Model

**DOI:** 10.1371/journal.pone.0013122

**Published:** 2010-09-30

**Authors:** Aurelia Syngkon, Sridhar Elluri, Hemanta Koley, Pramod K. Rompikuntal, Dhira Rani Saha, Manoj K. Chakrabarti, Rupak K. Bhadra, Sun Nyunt Wai, Amit Pal

**Affiliations:** 1 Divisions of Pathophysiology, National Institute of Cholera and Enteric Diseases, Kolkata, West Bengal, India; 2 Division of Bacteriology, National Institute of Cholera and Enteric Diseases, Kolkata, West Bengal, India; 3 Division of Histology and Electron Microscopy, National Institute of Cholera and Enteric Diseases, Kolkata, West Bengal, India; 4 Department of Molecular Biology, Umeå Centre for Microbial Research (UCMR), Umeå University, Umeå, Sweden; 5 Infectious Diseases and Immunology Division, Indian Institute of Chemical Biology, Kolkata, West Bengal, India; University of Hyderabad, India

## Abstract

**Background:**

Two well-characterized proteases secreted by *Vibrio cholerae* O1 strains are hemagglutinin protease (HAP) and *V. cholerae* protease (PrtV). The *hapA* and *prtV* knock out mutant, *V. cholerae* O1 strain CHA6.8Δ*prtV,* still retains residual protease activity. We initiated this study to characterize the protease present in CHA6.8Δ*prtV* strain and study its role in pathogenesis in rabbit ileal loop model (RIL).

**Methodology/Principal Findings:**

We partially purified the residual protease secreted by strain CHA6.8Δ*prtV* from culture supernatant by anion-exchange chromatography. The major protein band in native PAGE was identified by MS peptide mapping and sequence analysis showed homology with a 59-kDa trypsin-like serine protease encoded by VC1649. The protease activity was partially inhibited by 25 mM PMSF and 10 mM EDTA and completely inhibited by EDTA and PMSF together. RIL assay with culture supernatants of strains C6709 (FA ratio 1.1+/−0.3 n = 3), CHA6.8 (FA ratio 1.08+/−0.2 n = 3), CHA6.8Δ*prtV* (FA ratio 1.02+/−0.2 n = 3) and partially purified serine protease from CHA6.8Δ*prtV* (FA ratio 1.2+/−0.3 n = 3) induced fluid accumulation and histopathological studies on rabbit ileum showed destruction of the villus structure with hemorrhage in all layers of the mucosa. RIL assay with culture supernatant of CHA6.8Δ*prtV*ΔVC1649 strain (FA ratio 0.11+/−0.005 n = 3) and with protease incubated with PMSF and EDTA (FA ratio 0.3+/−0.05 n = 3) induced a significantly reduced FA ratio with almost complete normal villus structure.

**Conclusion:**

Our results show the presence of a novel 59-kDa serine protease in a Δ*hapA*Δ*prtV V. cholerae* O1 strain and its role in hemorrhagic response in RIL model.

## Introduction

Proteases are enzymes that catalyze the hydrolysis of peptide bonds in proteins or peptides. They are either exopeptidases, whose actions are restricted to the N-or C- termini of proteins, or endopeptidases which cleave internal peptide bonds. Microbial peptides are predominantly secreted enzymes and can be classified based on the essential catalytic residue at their active site. They include serine proteases, cysteine proteases, aspartate proteases and metalloproteases. Proteases produced by pathogenic microorganisms play an important role in virulence [1]. Tissue barriers to pathogen invasion, such as extracellular matrices, epidermal keratinocyte layers and blood vessel walls, may be targeted by bacterial proteases. Proteolysis of host tissue components such as extracellular matrix proteins, including collagen, laminin, fibronectin and elastin, can induce necrotic tissue damage [2], [3]. *V. cholerae* O1, the causative agent of epidemic cholera, secretes a 32-kDa zinc-containing hemagglutinin protease that may play a role in the pathogenesis of cholera. *V. cholerae* secretes hemagglutinin/protease (HAP), which is encoded by the *hap* gene [4], [5]. HAP can perturb the paracellular barrier function in epithelial cells by degrading occludin in tight junctions [6], [7]. HAP nicks the cholera toxin [8] and digests proteins, such as mucin, fibronectin, lactoferrin, and secretory immunoglobulin A, that may participate in host defense against cholera [9]. HAP can also hydrolyze mucin to enhance the detachment of *V. cholerae* from cultured epithelial cells [10]. A CTXφ- and *hap*-defective vaccine strain, 638, was not reactogenic in human volunteers and induced lower levels of IL-8 than its parent wild-type strain in HT29 cells [11]. The protease activity in *V. cholerae* vaccine strains reduced the transcellular epithelial resistance of polarized T84 intestinal epithelial cells [6]. These results suggested a role of HAP in reactogenicity, including inflammatory diarrhea. In our earlier studies, we have reported that HAP may play a role in the pathogenesis of a *ctx*-negative *V. cholerae* non-O1, non-O139 strain by inducing a hemorrhagic fluid response in the RIL assay [12]. Histopathological examination of 20 µg of purified protease-treated rabbit ileum showed the presence of erythrocytes and neutrophils in the upper part of the villus lamina propria [12].

Although HAP is a very active virulence factor, an isogenic strain of *V. cholerae* mutated in the *hap* gene was no less virulent in infant rabbits than the parental strain [13]. Fullner et al reported that a *hap* mutant is more lethal in a pulmonary murine model and caused more severe histopathological damage than its wild-type parent in the lungs of survivors, although no difference was seen in the induction of inflammation [14]. Studies by Zhou et al suggest that an IL-8 stimulator other than HAP may be responsible for inflammation contributing to the reactogenicity of attenuated *V. cholerae* vaccine strains [15]. An earlier study by Hase et al., [5] showed that a *hapA*-deleted mutant of *V. cholerae* O1 had reduced extracellular proteolytic activity compared with the parental strain in a skim milk assay, indicating that the mutant still produces some extracellular proteolytic activity. In addition, residual proteolytic activity expressed by the *hapA*-deleted mutant is distinct from HAP, as demonstrated by failure of anti-HAP serum to inhibit the activity of this secondary protease on milk agar. The mutant strain also failed to agglutinate chicken erythrocytes [5]. Young and Broadbent [16] described several extracellular proteases in *V. cholerae* that could explain the residual proteolytic activity of the *hap*-negative *V. cholerae* mutant. Besides HAP, the other major well- characterized protease in *V. cholerae* is a 97-kDa *Vibrio cholerae protease*, PrtV. PrtV plays a role in virulence in a *C. elegans* infection model [17].

In the present study, a *hap and prtV* double knock out mutant of *V. cholerae* strain CHA6.8Δ*prtV* still had residual protease activity. This protease was partially purified from strain CHA6.8Δ*prtV* and MS peptide mapping and sequence analysis of the protein revealed homology with a 59-kDa trypsin-like serine protease encoded by VC1649. To our knowledge, this is the first report of a serine protease in *V. cholerae* O1 and demonstration of its role in hemorrhagic response in the RIL model.

## Materials and Methods

### Ethics statement

Animal experiments were done after obtaining necessary permission from Institutional Animal Ethical Committee (IAEC). The IAEC/CPCSEA approval number is 45/1 dated 15/3/2007.

### Bacterial strains, plasmids and primers used in this study

The bacterial strains used in this study and their relevant properties are summarized in Table 1. All the strains were maintained at −80°C in 30% glycerol in tryptic soy broth (TSB, Difco laboratories). For protease purification, a Δ*hapA*Δ*prtV* mutant of *V. cholerae* O1 CHA6.8Δ*prtV* was used. Antibiotics were used at the following concentrations unless otherwise indicated: ampicillin (Am), 100 µg/ml; streptomycin (Sm), 100 µg/ml; carbenicillin (Cb), 100 µg/ml; kanamycin (Km), 50 µg/ml for *E. coli* and 40 µg/ml for *V. cholerae*.

**Table 1:** Bacterial strains, plasmids, primers and oligonucleotides used in this study.

Strains	Relevant genotype or phenotype	Source or Reference
*Vibrio cholerae*		
C6709	Wild-type (O1 El Tor); Sm^r^	[38]
CHA6.8	C6709Δ*hapA::kan*; Sm^r^, Km^r^	This study
CHA6.8Δ*prtV*	CHA6.8Δ*prtV*; Sm^r^, Km^r^	This study
CHA6.8Δ*prtV*ΔVC1649	CHA6.8Δ*prtV*ΔVC1649; Sm^r^, Km^r^	This study
*E. coli*		
DH5α	F' *endA1 hsdR17 supE44 thi-1 recA1 gyrA96 relA1* Δ (*argF*-*lacZYA*) U169 (Φ80*dlacZ* Δ*M15*)	Promega
SM10λpir	*thi thr leu tonA lacY supE recA*::RP4-2-Tc::Mu λ*pir R6K*	[38]
**Plasmids**		
PCR®4-TOPO®	pUC ori, high copy number cloning vector; Amp^r^ Km^r^	Invitrogen
pUC4K	Source of the kanamycin gene cassette; Amp^r^ Km^r^	Pharmacia
pKAS32	*rpsL* suicide vector with *ori*R6K *mob*RP4; Amp^r^	[18]
pHA1.8	1.8-kb PCR amplified *hapA* gene of *V. cholerae* strain C6709 in PCR®4-TOPO®; Ap^r^, Km^r^	This study
pHA2.4	PCR®4-TOPO® containing 2.4-kb ΔhapA::kan allele; Ap^r^, Km^r^	This study
pHA6.8	Suicide vector pKAS32 containing 2.4 kb Δ*hapA::kan* allele; Ap^r^, Km^r^	This study
pJZ215	Suicide vector pJZ215:: Δ*prtV*; Cb^r^	This study
pCVD442	Suicide vector pCVD442:: ΔVC1649; Cb^r^	This study
**Oligonucleotides**		
Hap-F1	5′-AATACGGCAGTAACGGTTTA-3′	This study
Hap-F2	5′-CAACGTCCTCTGAATTGGT-3′	This study
Hap-R1	5′-CGTAACGTCACACCAGAATA-3′	This study
PrtV-4	5′-GAAGGAAGAAGCGGCCGCCATTTTATTTCCTTAATATTTCCTT-3′	This study
PrtV-1	5′-GTTGACTCGAGTTACCG-3′	This study
PrtV-3	5′-CGGGATC-CGTTATATCGCCAGCATG-3′	This study
PrtV-2	5′-GAAATAAAATGGCGGCCGCTTCTTCCTTCTCCTTCCATGGATT -3′	This study
VC1649-A	5′-CGCTCTAGAGTAACAAGCTTGTGTAGCCAC-3′	This study
VC1649-B	5′-CCCATCCACTATAAACTAACAAGCGTTCCAGAAGCACTGAACTG3′	This study
VC1649-C	5′-TGTTAGTTTATAGTGGATGGGCCGCTTGATATTCGTATCGGT-3′	This study
VC1649-D	5′-CGCTCTAGAGGATGAAACCTTGGTACTGAC-3′	This study
**Internal Primers**		
Hap-F	5′-GTGAACAACACGCTGGAGAA-3′	This study
Hap-R	5′-CGTTGATATCCACCAAAGG-3′	This study
PrtV-F	5′-CATACTGAGATGCTCTACGAT-3′	[17]
PrtV-R	5′-TTTCACCATGTTCGGGCGTGA-3′	[17]
VC1649-F	5′-GGTGGTAGTTATCTTGGTGG-3′	This study
VC1649-R	5′-GTCACAACTCGCTCCTGAA-3′	This study
Ctx A- F	5′-CGGGCAGATTCTAGACCTTCCTG-3′	[37]
Ctx A- R	5′-CGATGATCTTGGAGCATTCCCAC-3′	[37]
**Sequencing primers**		
VC1649 -F1	5′-CCGTTCATACTCTGCAATAG-3′	This study
VC1649 -R1	5′-CAGCAGTCAAAACATAACGACCACC-3′	This study
VC1649 -F2	5′-GCTTCAATGGGCAATGATATTGCTG-3′	This study
VC1649 -F3	5′-GTCGAAAGCAGATGGGCGTTGTTAG-3′	This study
VC1649 -F4	5′-GGATCAAGTACTTATAAGACGGGTGC-3′	This study
VC1649- F5	5′-CAGAAGACATAGCGGTAGTACTGATG-3′	This study
VC1649 -R2	5′-CACGGTTGGCCTCGAGTAAACAAAATTGGC-3′	This study

### Construction of a *hapA* knock -out mutant in *Vibrio cholerae* O1 strain C6709

The bacterial mutant was constructed by replacing the *hapA* gene with its deletion-insertion allele Δ*hapA*::*kan* using published methods [18]. One such mutant showing a Sm^r^ Km^r^ phenotype was selected for further study and designated CHA6.8 (Table 1). The in-frame *hapA* gene deletion in the strain C6709 was confirmed by PCR with *hapA* internal primers (Table 1).

### Construction of a Δ*hapA*Δ*prtV V. cholerae* O1 strain, CHA6.8Δ*prtV*

The bacterial mutant was constructed by double crossover method using the construct as mentioned previously [17]. The knock out mutant (CHA6.8Δ*prtV*) was confirmed with *prtV* internal primers (Table 1).

### Construction of Δ*hapA*Δ*prtV*ΔVC1649 *V. cholerae* O1 strain CHA6.8Δ*prtV*ΔVC1649

A VC1649 in-frame deletion mutant was constructed using published methods [18], [19]. Several colonies were purified from the plates, tested for Cb sensitivity and then analyzed for the deletion and confirmed with internal primers for *hapA*, *prtV*, *VC1649* and *ctx* (Table 1).

### Azocasein assay

Casein was chosen as the substrate to assay proteolytic activity. The substrate-enzyme mixture was incubated at 37°C and the reaction was terminated with 10% trichloroacetic acid after 1 hour. The precipitated protein was removed by centrifugation (12,000× g for 4 mins), and the supernatant was transferred to a clean tube containing 525 mM NaOH. Absorbance was measured at 440 nm using a Smartspec spectrophotometer (Bio-Rad, Hercules, CA). Substrate with buffer and substrate with inhibitors were used as negative controls.

### Skim milk assay

Single colonies of *V.cholerae* O1 strains C6709, CHA6.8, CHA6.8Δ*prtV* and CHA6.8Δ*prtV*ΔVC1649 were streaked onto nutrient agar (NA) plates containing 1.5% skim milk and incubated at 37°C overnight. Protease activity was detected by clearing of the opaque milk proteins incorporated into the NA.

### Inhibition of protease activity

The azocasein assay was done with 30 µg of ammonium sulphate precipitated proteins from culture supernatants of C6709, CHA6.8, CHA6.8Δ*prtV* and CHA6.8Δ*prtV*ΔVC*1649.* The protease activity of crude proteins was also tested for inhibition with 25 mM PMSF, 10 mM EDTA and 10 mM 1,10- phenanthroline. Twenty-five mM Tris-HCl and 25 mM Tris-HCl in the presence of 25 mM PMSF, 10 mM EDTA and 10 mM 1,10- phenanthroline were used as negative controls. The NB (non-binding) fraction (5 µg) of the CHA6.8Δ*prtV* strain with protease inhibitors (10 mM EDTA, 10 mM EGTA, 25 mM PMSF, 10 mM EDTA with 25 mM PMSF, 1 µg/ml aprotinin, 28 mM E-64, 1 µg/ml leupeptin and 10 mM 1,10- phenanthroline) were incubated for 30 mins at 37°C and assayed by azocasein assay. Twenty-five mM Tris-HCl was used as a negative control. The protease activity with EDTA was measured both in the presence and absence of 10 mM CaCl_2_. The mean with standard deviation of three experiments was considered for data analysis.

### Partial purification of a novel protease from strain CHA6.8Δ*prtV*

The Δ*hapA*Δ*prtV V. cholerae* O1 strain CHA6.8Δ*prtV* was grown in 3 l of tryptic soy broth (TSB) for 18 h under agitation in an orbital shaker (OSI503; Firstek Scientific). Cells were harvested by centrifugation at 8,000× g for 20 min at 4°C in a SS34 rotor (Sorvall, Newtown, Connecticut). The protein in the cell-free culture supernatant was precipitated with 60% saturated ammonium sulphate. After centrifugation at 11,973× g for 20 min at 4°C, the pellet was re-suspended in 25 mM Tris-HCl buffer, pH 7.4. Re-suspended proteins were dialyzed against the same buffer, concentrated by Amicon filtration (Millipore Co, Bellerica MA) and loaded onto an ion exchange chromatography column (DE-52; Whatman, Kent, UK) pre-equilibrated with 25 mM Tris-HCl buffer, pH 7.4. Proteins eluted in the unbound fraction were designated as the non-binding fraction (NB). The proteins bound to the DE-52 column were eluted in the presence of NaCl (0.1 and 0.3 M). Fractions constituting the peaks NB, 0.1 M #1, 0.1 M #2 and 0.3 M were pooled, dialyzed, concentrated and examined for protease activity by azocasein assay. The columns were run on a BioLogic Duo Flow Chromatographic system (Bio-Rad, Hercules, CA).

### Native PAGE

The proteins were separated by electrophoresis on a 10% native polyacrylamide gel according to the procedures described by Davis et al [20] in the absence of SDS and 2-mercaptoethanol. Protein samples were mixed with sample buffer containing 10% glycerol, 0.05% bromophenol blue and Tris-HCl pH-6.8, resolved in the gel and bands were visualized by staining with Coomassie brilliant blue.

### Protein identification by MS peptide mapping and sequencing analysis

The major band observed in the native PAGE of the non-binding pooled fraction from the DE-52 column was excised from the Coomassie blue stained gel and analysed on a Bruker Autoflex III MALDI TOF/TOF instrument at Alphalyse, Odense, Denmark. The peptide mixture was analyzed in positive reflector mode for accurate peptide mass determination and 5–10 of the peptides were selected for analysis by MS/MS fragmentation for partial peptide sequencing. The MS and MS/MS spectra were combined and used for a database search in an in-house protein database using the Mascot software. The peptides used for the identification are highlighted in the sequence. Peptides confirmed by MS/MS sequencing are shown in bold.

### DNA sequencing

The VC1649 gene (ORF) was amplified by using primers 5′CCGTTCATACTCTGCAATAG3′and5′CACGGTTGGCCTCGAGTAAACAAAATTGGC3′. The resultant PCR product was analyzed by 0.7% agarose gel electrophoresis. The sequencing was done on an ABI 3130 DNA analyzer (Applied Biosystems, Foster City, CA) and the sequences were aligned, analyzed using Clustal X and NCBI/BLAST programs. The nucleotide sequence data reported in this paper will appear in the DDBJ/EMBL/GenBank nucleotide sequence database with the accession number AB572560.

### Rabbit ileal loop assay

The rabbit ileal loop (RIL) assay was performed in young New Zealand White rabbits (2 kg) essentially by the method described by De and Chatterjee [21]. Culture supernatants (one ml) and washed bacterial cells (10^9^cfu/ml) of C6709, CHA6.8, CHA6.8Δ*prtV*, CHA6.8Δ*prtV*ΔVC1649 grown in tryptic soy broth were inoculated in rabbit ileum. Tryptic soy broth was used as negative control in the above assay. The partially purified protease at a concentration of 50 µg (non-binding pooled fraction eluted from DE-52 column) from CHA6.8Δ*prtV* strain, similar concentration of protease inhibited with 25 mM PMSF and EDTA and 25 mM Tris-HCl with PMSF and EDTA (negative control) in a volume of 1 ml were also assayed in RIL assay. The animals were sacrificed after 18 hrs and the enterotoxic response was determined by measuring the fluid accumulation (FA) ratio, which is the ratio of the volume of fluid accumulated in the intestinal loop to the length of the loop. A ratio of greater than 1.0 indicated a strong positive response, while a negative response was defined as FA ratio of less than 0.2.

### Histopathological studies

Tissue samples (2 cm in length) from RIL assays were collected and placed in 10% neutral-buffered formalin for histopathological analysis. Tissues were embedded in paraffin and processed following the standard protocol. Sections (3 to 4 µm thick) prepared with a Leica rotary microtome were stained with hematoxylin and eosin and examined by light microscopy. Photographs were taken under different magnifications with a Leica DMLB microscope (Solms, Germany), equipped with a digital imaging system.

## Results

### Construction of Δ*hapA* and Δ*hapA*Δ*prtV V. cholerae* O1 mutant strains

The in-frame *hapA* deletion in the strain C6709 was confirmed by PCR using internal primers (Table 1). The CHA6.8Δ*prtV* mutant was constructed by the double crossover method as described previously (Vaitkevicius et al 2006). The knock out mutant CHA6.8Δ*prtV* was confirmed using *prtV* specific internal primers (Table 1).

### Protease activity in C670*9,* CHA6.8 and CHA6.8Δ*prtV* strains

**Figure 1:**
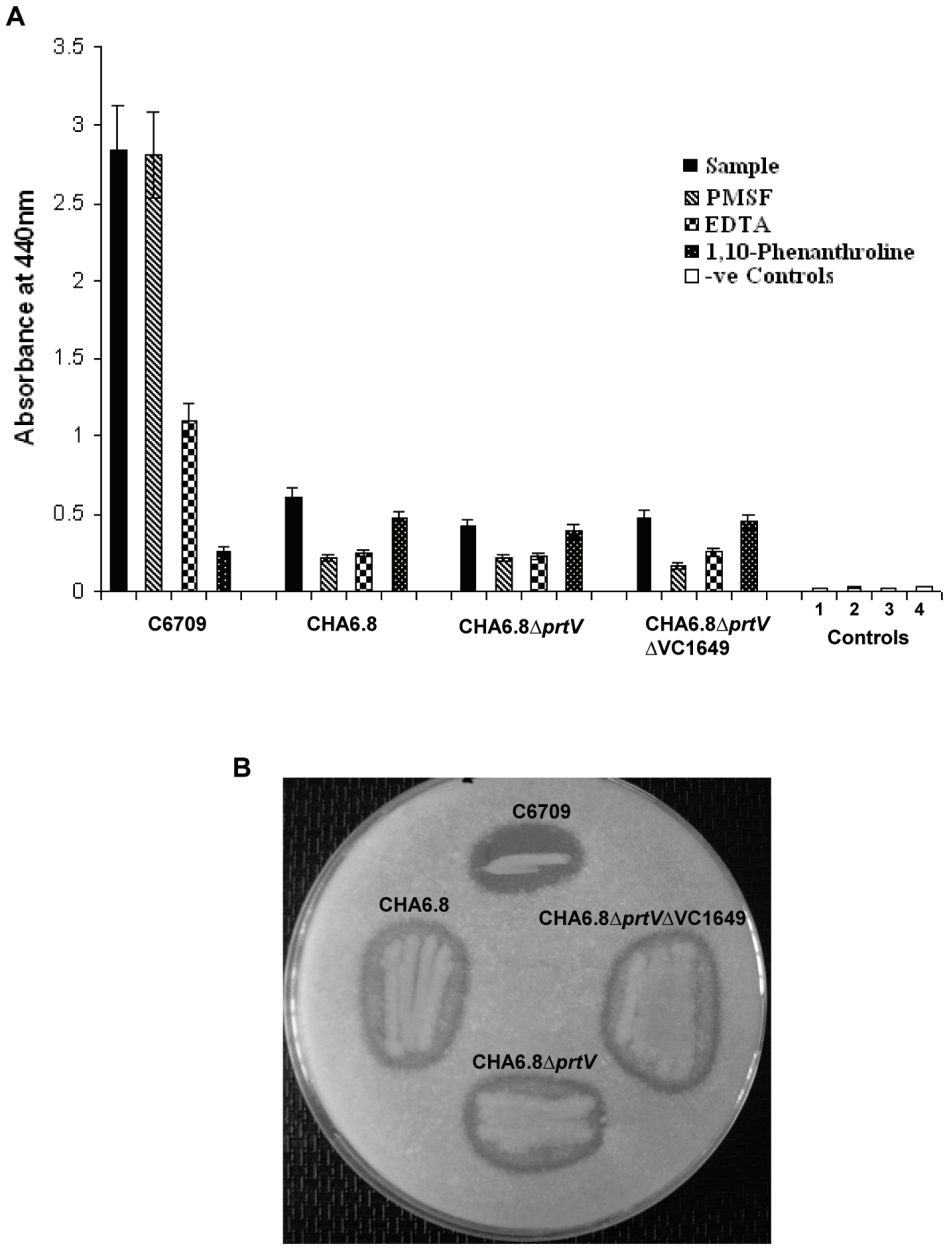
Protease activity assay. A) Azocasein assay with 30 µg of ammonium sulphate precipitated proteins from culture supernatants of C6709, CHA6.8, CHA6.8Δ*prtV* and CHA6.8Δ*prtV*ΔVC*1649* and inhibition test with 25 mM PMSF, 10 mM EDTA and 10 mM 1,10- phenanthroline. Negative controls were (1) 25 mM Tris-HCl and 25 mM Tris-HCl in the presence of (2) 25 mM PMSF, (3) 10 mM EDTA and (4) 10 mM 1,10- phenanthroline. The values shown are the means with standard deviations from three experiments. B) Skim milk assay for detection of protease in C6709, CHA6.8, CHA6.8Δ*prtV* and CHA6.8Δ*prtV*ΔVC*1649* strains.

The proteins from culture supernatants of the above strains were precipitated with ammonium sulphate and after dialysis, 30 µg of crude proteins were tested for protease activity by azocasein assay. As shown in Fig. 1A, *V*. *cholerae* strain C6709 had maximum protease activity compared to the other strains. The protease activity in strain C6709 was inhibited in the presence of EDTA and 1, 10- phenanthroline. 1, 10-phenanthroline, a specific metalloprotease inhibitor, inhibited 90.7% of the protease activity, confirming that the major protease activity in this strain is due to metalloproteases (Fig. 1A). EDTA, a non-specific metalloprotease inhibitor, repressed 61% of the protease activity (Fig. 1A). PMSF, a serine protease inhibitor, did not change the protease activity, suggesting that there is no serine protease activity in C6709 (Fig. 1A). *V. cholerae* O1 strain CHA6.8, in which the *hapA* gene has been knocked out, had a 78.7% decrease in protease activity when compared to strain C6709. The protease activity of crude proteins from CHA6.8 in the presence of 1, 10-phenanthroline decreased by 21.1%, indicating the presence of other metalloprotease in the absence of HAP in strain CHA6.8. Both EDTA and PMSF significantly decreased protease activity in strain CHA6.8 by 58.9% and 63.9%, respectively. The major protease present in strain CHA6.8 is a serine protease, although metalloproteases are also present. Besides HAP, the other well-characterized metalloprotease in *V. cholerae* is the 97-kDa *V. cholerae* protease (PrtV). The Δ*hap* and Δ*prtV V. cholerae* O1 strain CHA6.8Δ*prtV* had decreased protease activity by 29.6% compared to strain CHA6.8, indicating that PrtV also contributes to the protease activity of strain CHA6.8 (Fig. 1A). Both EDTA and PMSF significantly decrease protease activity, by 48.7% and 47%, respectively, suggesting that the major protease activity in the CHA6.8Δ*prtV* strain is due to a serine protease. The 48.7% decrease in CHA6.8Δ*prtV* due to EDTA is not due to a metalloprotease, as 1, 10- phenanthroline, a specific metalloprotease, only reduced protease activity by 8.4% (Fig. 1A).

All strains included in this study were also tested for protease activity in a skim milk assay. As shown in Fig. 1B, the zone of proteolysis created by C6709 on skim milk agar was clear, indicating complete degradation of the milk proteins. With the other two strains, CHA6.8 and CHA6.8Δ*prtV*, the zone of proteolysis was hazy, indicating that not all of the milk proteins were degraded. These results suggest that the substrate specificity of proteases in CHA6.8, and CHA6.8Δ*prtV* may be different from that of C6709, which secretes HAP.

### Partial purification of protease from the strain CHA6.8Δ*prtV*

**Figure 2:**
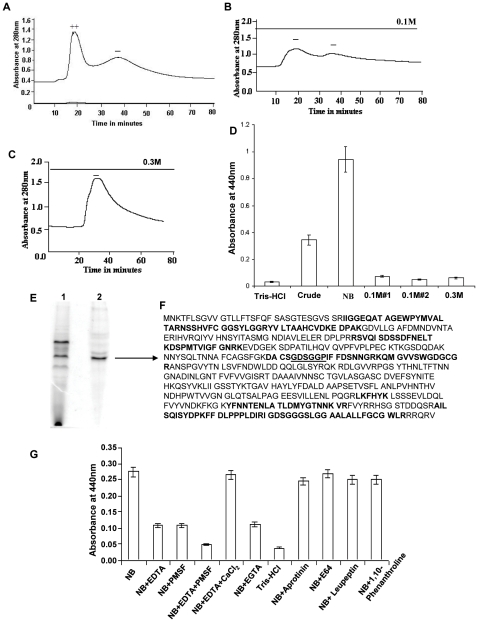
Partial purification and identification of protease. Chromatographic profile of ammonium sulphate precipitated crude proteins from culture supernatants of CHA6.8Δ*prtV* strain loaded onto an anion exchange column (DE-52). A) Proteins eluted in the non-binding fraction (NB), B) proteins eluted with 0.1 M NaCl, C) proteins eluted with 0.3 M NaCl, +/− shows presence or absence of protease activity, D) azocasein assay with pooled samples (30 µg) NB, 0.1 M#1, 0.1 M #2, 0.3 M and crude proteins. E) Native PAGE profile (lane 1) of crude proteins of CHA6.8Δ*prtV* strain and (lane 2) of partially purified protease (NB) from DE-52 column. The marked protein band was analyzed by MS/MS sequencing and the peptides highlighted showed homology with a 59-kDa trypsin-like serine protease encoded by VC1649. F) The underlined GDSGGP are the amino acid sequences around the serine residue present in trypsin-like serine proteases. G) Protease inhibition test of NB fraction (5 µg) with protease inhibitors 10 mM EDTA, 25 mM PMSF, 25 mM PMSF and 10 mM EDTA, 10 mM EDTA and 20 mM CaCl_2_, 10 mM EGTA, 1 µg/ml aprotinin, 28 mM E64, 1 µg/ml leupeptin and 10 mM 1,10-phenanthroline incubated for 30 mins at 37°C. Residual protease activity was assayed by azocasein assay. Twenty-five mM Tris-HCl was used as a negative control. The values shown are the means with standard deviations from three experiments.

The ammonium sulphate precipitated proteins from culture supernatants of CHA6.8Δ*prtV* were loaded onto an anion-exchange chromatography column (DE-52). The proteins in the non-binding fraction of the column (Fig. 2A) were pooled and concentrated. The bound proteins were eluted with 0.1 M (Fig. 2B) and 0.3 M (Fig. 2C) NaCl, dialyzed against 25 mM Tris-HCl buffer and concentrated. When protease activity in the NB, 0.1 M and 0.3 M NaCl eluted fractions were tested by azocasein assay, the major protease activity was present in the NB fraction (Fig. 2D). The NB-pooled fraction was concentrated and run on a native PAGE (Fig. 2E). The major protein band was excised and analyzed by MS/MS sequencing (Fig. 2E). The sequences highlighted showed homology with a 59-kDa serine protease encoded by the gene VC1649 (Fig. 2F). The sequence GDSGGP (underlined) flanks the serine residue in trypsin-like serine proteases (Fig. 2F).

### The presence of a calcium-dependent serine protease

To determine the nature of the partially purified protease from CHA6.8Δ*prtV* eluted in the non-binding fraction of a DE52 anion-exchange column, we performed protease inhibition assays with several inhibitors (Fig. 2G). The protease was partially inhibited in the presence of EDTA (60.3%), EGTA (59.2%) and PMSF (60.3%). The partially purified protease was completely inhibited when PMSF and EDTA are used together (Fig. 2G). There was significantly less inhibition of protease activity in the presence of 1,10- phenanthroline (9%), aprotinin (10.5%), leupeptin (8.7%) and E64 (1.8%). Although EDTA inhibited protease activity by 60.3%, EDTA in the presence of CaCl_2_ inhibited activity by only 3.6% inhibition (Fig. 2G). The serine protease secreted by CHA6.8Δ*prtV* is a calcium-dependent serine protease.

### Construction of CHA6.8Δ*prtV*ΔVC1649 deletion mutant

**Figure 3:**
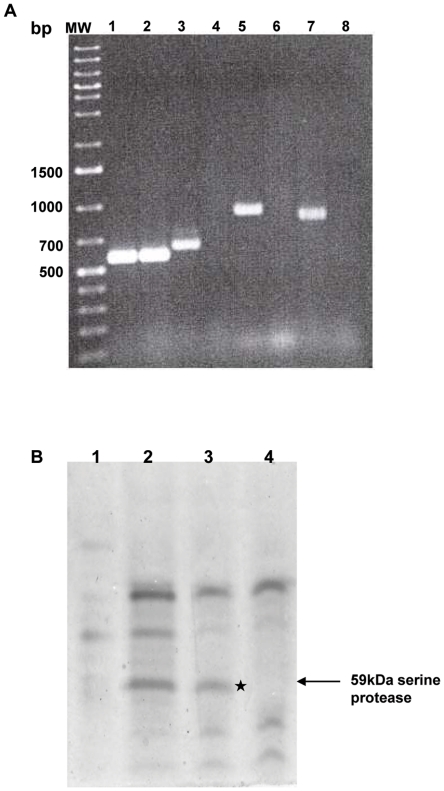
Confirmation of knock out mutant. A) PCR amplification with internal primers for *ctx* (lane 1), *hapA* (lane 3), *prtV* (lane 5) and VC1649 (lane 7) in strain C6709 and for *ctx* (lane 2), *hapA* (lane 4), *prtV* (lane 6) and VC1649 (lane 8) in strain CHA6.8Δ*prtV*ΔVC1649. The primer sequence used in the above experiment is given in Table 1. (MW) denotes molecular weight marker (20 kb-75 bp marker, Fermentas). B) Native PAGE (10%) with ammonium sulphate precipitated proteins of C6709 (lane 1), CHA6.8 (lane 2), CHA6.8Δ*prtV* (lane 3) and CHA6.8Δ*prtV*ΔVC1649 (lane 4). The * shows the protein band with sequence homology to the 59-kDa serine protease (VC1649). This band is absent in strain CHA6.8Δ*prtV*ΔVC1649 (lane 4).

The CHA6.8Δ*prtV*ΔVC1649 deletion mutant was constructed as described in the text and confirmed by internal primers for *hapA, prtV* and VC1649 as shown in Fig. 3A. PCR with internal primers for the *ctx* gene confirmed the presence of *ctx* in both the C6709 and the CHA6.8Δ*prtV*ΔVC1649 strains. PCR with internal primers for *hapA, prtV* and VC1649 in strain CHA6.8Δ*prtV*ΔVC1649 showed the absence of any band by agarose gel electrophoresis (Fig. 3A) and also confirmed the absence of the deleted gene sequences. On the other hand, strain C6709 showed the presence of PCR products with the internal primers confirming the presence of *hapA, prtV* and VC1649 genes (Fig. 3A). A native PAGE profile of crude proteins from CHA6.8Δ*prtV* and CHA6.8Δ*prtV*ΔVC1649 showed the absence of a 59-kDa band in the CHA6.8Δ*prtV*ΔVC1649 strain (Fig. 3B). The VC1649 gene sequence showed complete homology with the published sequence of VC1649 from *V. cholerae* O1 biovar El Tor strain N16961 (NCBI).

We initially started the study to characterize the proteases present in the CHA6.8Δ*prtV* strain. Partial purification of proteases from CHA6.8Δ*prtV* strain showed the presence of a 59-kDa trypsin-like serine protease encoded by the VC1649 gene. The major protease present in the CHA6.8Δ*prtV* strain is a serine protease, but protease activity in CHA6.8Δ*prtV*ΔVC1649, in which the 59-kDa serine protease is not secreted, increased by 11.4% (Fig. 1A). These results indicated that besides HAP, PrtV and a 59-kDa serine protease, there are still other proteases secreted by the CHA6.8Δ*prtV*ΔVC1649 strain. PMSF and EDTA inhibited protease activity in CHA6.8Δ*prtV*ΔVC1649 by 64.5% and 46.2%, respectively, whereas 1,10-phenanthroline only inhibited protease activity by 6.2% suggesting the presence of another serine protease (Fig. 1A). Skim milk assay with CHA6.8Δ*prtV*ΔVC1649 still showed residual protease activity (Fig. 1B).

### The serine protease induces a hemorrhagic fluid response in RIL

**Figure 4:**
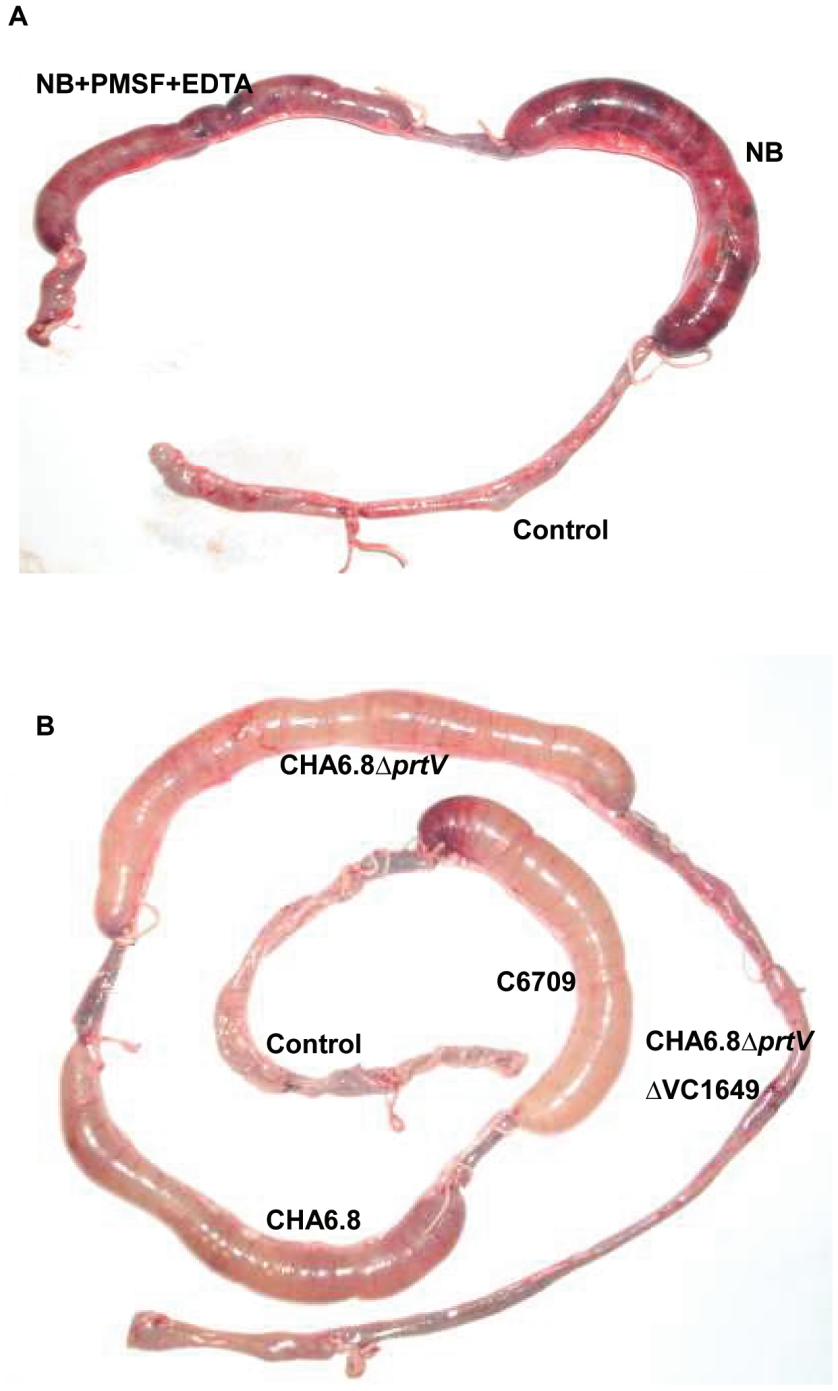
Rabbit ileal loop assay. A) RIL response of partially purified protease (50 µg, NB) showing significant hemorrhagic fluid accumulation (FA ratio 1.2+/−0.2 n = 3) and its effect after inhibition with 25 mM PMSF and 10 mM EDTA (NB+PMSF+EDTA) shows significant decrease in fluid accumulation (FA ratio 0.3+/−0.05 n = 3). Twenty five mM Tris-HCl with 25 mM PMSF +10 mM EDTA was used as a negative control (FA ratio = 0.12+/−0.002, n = 3). B) RIL response with culture supernatants of C6709 (FA ratio 1.1+/−0.3, n = 3), CHA6.8 (FA ratio 1.08+/−0.2, n = 3), CHA6.8Δ*prtV* (FA ratio 1.02+/−0.2, n = 3), CHA6.8Δ*prtV*ΔVC1649 (FA ratio 0.11+/−0.005, n = 3) and Tryptic soy broth as negative control (FA ratio 0.09+/−0.002, n = 3).

**Figure 5:**
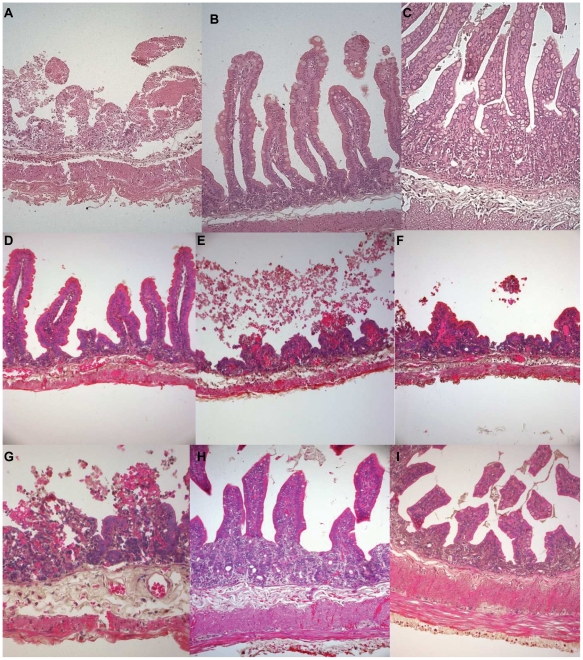
Histopathological study of ileal tissues. Panels show photomicrographs of histology of rabbit ileal loop tissue after treatment with A) Partially purified serine protease from *V. cholerae* strain CHA6.8Δ*prtV* showing hemorrhagic fluid accumulation (Fig. 4A, NB). Gross damage of the villus surface structure was observed with hemorrhage in all layers of the mucosa. Magnification, 20X. B) Almost normal villous architecture observed in ileal tissues treated with 50 µg of partially purified protease inhibited with 25 mM PMSF and 10 mM EDTA (Fig. 4A, NB+PMSF+EDTA). This photomicrograph shows no gross alteration in villus structure but villus lamina propria are slightly dilated and RBC have accumulated at a few places in the basal area. Magnification, 20X. C) Ileal tissues treated with 25 mM Tris-HCl buffer with PMSF and EDTA (Fig. 4A, control) showed normal villus structure. Magnification 20X. (D) ileal tissues treated with culture supernatant from C6709 strain showed presence of hemorrhage in all layers of the gut mucosa specially in the submucosal layer, Magnification 20X. E) ileal tissues treated with culture supernatant from CHA6.8 strain showed widely dialated villi with rupture at places with gross hemorrhage and inflammatory cells in mucosa and submucosa, Magnification 20X. F) Ileal tissues treated with culture supernatant of CHA6.8Δ*prtV* strain also showing dilated villi with gross hemorrhage in all layers of the mucosa. Magnification 20X. G) The same section in higher magnification 40X showing ruptured villi with hemorrhage and inflammatory cells in mucosa and submucosa. (H) ileal tissues treated with culture supernatant from CHA6.8Δ*prtV*ΔVC1649 strain showing villous architecture almost normal with minimum hemorrhage in mucosa and submucosa. (I) TSB treated ileal tissue showing normal gut mucosa.

To study the role of the 59-kDa serine protease in virulence, 50 µg of the partially purified protease was injected into the rabbit ileum, which induced significant hemorrhagic fluid accumulation (FA ratio 1.2+/−0.2, n = 3, Fig. 4A). When a similar concentration of the protease was incubated in the presence of PMSF and EDTA and injected into the rabbit ileum, there was a significant decrease in fluid accumulation (FA ratio 0.3+/−0.05, n = 3, Fig. 4A). Histopathological analysis of the rabbit ileum revealed that the protease caused extensive damage to all the layers of the mucosa. There was damage to the villus structure, which was completely destroyed. We observed gross damage of the villus surface structure with hemorrhage in all layers of the mucosa (Fig. 5A). On the other hand, analysis of the ileal tissues treated with the protease in presence of both PMSF and EDTA, revealed normal microvillus structure with no gross alteration in villus structure, although the villus lamina propria was slightly dilated and RBCs had accumulated in a few places in the basal area (Fig. 5B). PMSF and EDTA completely inhibited protease activity (Fig. 4A), but still we observed some residual effect in the rabbit ileal loop. This effect could be due to some other domain in the protease, which may not be its proteolytic domain, and could be responsible for causing damage to the ileal tissue. Tissues treated with 25 mM Tris-HCl and PMSF + EDTA did not cause fluid accumulation in RIL (FA ratio 0.12+/−0.002 n = 3, Fig. 4A) and histopathology of the ileal tissue showed normal microvillus structure (Fig. 5C).

One ml of culture supernatant of C6709, CHA6.8 and CHA6.8Δ*prtV* strain induced significant fluid accumulation (Fig. 4B) in RIL model (FA ratio 1.1+/−0.3, n = 3; 1.08+/−0.2, n = 3; and 1.02+/−0.2, n = 3 respectively) where as CHA6.8Δ*prtV*ΔVC1649 strain which is devoid of the serine protease gene and sterile tryptic soy broth, TSB (negative control) did not induce any fluid accumulation (Fig. 4B) (FA ratio 0.11+/−0.005, n = 3 and 0.09+/−0.002, n = 3 respectively). Almost similar results were observed when one ml of washed bacterial cells (10^9^ cfu/ml) of C6709, CHA6.8 and CHA6.8Δ*prtV* induced significant fluid accumulation (FA ratio 1.2+/−0.35, n = 3; 1.1+/−0.3, n = 3; and 1.0+/−0.2, n = 3 respectively). Bacterial cells of CHA6.8Δ*prtV*ΔVC1649 strain did not induce any fluid accumulation (FA ratio 0.15+/−0.005, n = 3). Histopathological studies of ileal tissues treated with culture supernatant from C6709 strain showed presence of hemorrhage in all layers of the gut mucosa especially in the sub-mucosal layer (Fig. 5D). Ileal tissues treated with culture supernatant from CHA6.8 strain showed widely dilated villi with rupture at places with gross hemorrhage and inflammatory cells in mucosa and sub-mucosa (Fig. 5E). Ileal tissues treated with culture supernatant of CHA6.8Δ*prtV* strain also showed dilated villi with gross hemorrhage in all layers of the mucosa (Fig. 5F). The same section at a higher magnification 40X showed ruptured villi with hemorrhage and inflammatory cells in mucosa and sub-mucosa (Fig. 5G). The ileal tissues treated with culture supernatant of CHA6.8Δ*prtV*ΔVC1649 strain protease showed villous architecture almost normal with minimum hemorrhage in mucosa and sub-mucosa (Fig. 5H). TSB treated ileal tissue showed normal gut mucosa (Fig. 5I).

## Discussion

Bacterial proteases are an important virulence factor in a variety of organisms, causing massive tissue damage which may aid the bacteria in host cell entry [22]. The major protease secreted by *V. cholerae* is HAP, a member of a large family of metalloproteases. HAP is produced by both non-pathogenic and pathogenic species, including the elastase of *P. aeroginosa* [23]. It acts on potentially relevant substrates like mucin, fibronectin, lactoferrin and the A subunit of CT [8], [10]. In our earlier studies, we demonstrated that HAP may play an important role in the pathogenesis of *ctx*-negative *V. cholerae* non-O1, non-O139 strains [12]. We have also shown that the processed 35-kDa form of HAP induces a dose-dependent hemorrhagic response in the RIL assay, a decrease in the intestinal short circuit current (Isc) in an Ussing chamber, and a cell rounding effect on HeLa cells. Fullner et al., reported that the deletion of *hap* in *V. cholerae* did not affect the production of IL-6, or macrophage inflammatory protein 2 in a murine pulmonary model, and the hap mutant was more virulent than its wild-type parental strain, although the mechanism was not clear [14]. When *V. cholerae hapA* mutants were tested in the *C. elegans* killing assay, *hapA* deleted strains were not attenuated compared to wild-type *V. cholerae* O1 [17]. The culture supernatant from a *hapA* mutant contained proteins bands encoded by the ORFs VCA0812, VCA0813, and VCA0223 [24], as determined by mass spectrometry. The protein products are a leucine aminopeptidase-related protein, leucine aminopeptidase (Lap) [25], and the PrtV protease [26], respectively. When Δ*prtV*, Δ*lap*, and Δ*lapX* mutants were tested in the *C. elegans* assay, the Δ*prtV* mutant was completely attenuated compared to the wild-type strain. The PrtV protein is a factor required for the *V. cholerae* lethal infection of *C. elegans* [17]. Earlier studies with *hapA* mutant strains showed that deletion of this gene still produces some extracellular proteolytic activity [5] Measurements of proteolytic activity against azocasein indicated that 10–20% of total activity in culture supernatants was abolished by the Δ*prtV* mutation [17]. In the same study, deletion of *hapA* reduced the total protease activity to 10% of the wild-type level. HAP, being the major protease in *V. cholerae* O1, could mask the other secretory proteases. As shown earlier, strain C6709 does not exhibit serine protease activity, but in absence of *hapA* the serine protease is secreted by strain CHA6.8. Our results suggest that the expression of proteases in *V. cholerae* may follow a cascade of events. HAP controls secretion of the 59-kDa serine protease, which in turn may control secretion of other proteases. Further experiments are being done to confirm these results.

The role of proteases other than HAP can best be studied in *hapA* mutant strains. The objective of our study was to identify the protease present in the Δ*hapA,* Δ*prtV V. cholerae* O1 strain CHA6.8Δ*prtV* and study the role of this protease in pathogenesis. The protease was partially purified and its activity was observed in the non-binding fraction of an anion exchange column. The major band of this partially purified protease, as visualized by native PAGE, was analyzed by mass peptide sequencing and found to be homologous to a trypsin-like serine protease encoded by the VC1649 gene. The serine protease also had the GDSGGP sequence normally associated with trypsin-like serine protease [27]. Interestingly, when the nature of this partially purified protease was studied using inhibitors, it was observed that EDTA, EGTA and PMSF could partially inhibit its protease activity. Protease activity was completely inhibited in the presence of PMSF and EDTA together. The specific metalloprotease inhibitors, like 1, 10-phenanthroline, could not inhibit the protease activity, nor could the other inhibitors like E-64, aprotinin, leupeptin and bestatin. EDTA with CaCl_2_ failed to inhibit protease activity. Our results showed that the protease present in the *hapA,prtV*-deleted strain is a calcium-dependent serine protease. In an earlier study by Young and Broadbent [16], 100 strains of *V. cholerae* El Tor from different parts of the world were screened for protease production by a rapid assay with gelatin agar plates. Based on protease production, the strains were classified as high, medium and low protease producers. Protease I activity (as shown by PMSF inhibition) was detected only in low protease producers, whereas protease II activity (marked stimulation by EDTA) was associated with the high protease producers. Protease III activity (EDTA inhibition) was difficult to detect in the presence of large amounts of protease II, but it was seen in some low protease producers. It is possible that the absence of protease I in the high protease producers is simply due to masking of this activity by the much larger amounts of protease II. Filtrates from the low protease producing strain 1621 contained predominantly type I protease activity, which is sensitive to serine protease inhibitors such as PMSF and the lima bean trypsin inhibitor. Activation of CT by limited proteolysis of the A subunit is also sensitive to serine protease inhibitors [28], [29], and it seems likely, therefore, that this involves the type I protease. These results were, however, carried out with strain 569B, a low protease producer in which type I protease is readily detectable. This report clearly suggests that in high protease producers, in which EDTA can inhibit activity, the protease could be hemagglutinin protease; in strains in which HAP is not secreted, the serine protease could be the major protease. The 59-kDa serine protease could be the major protease in classical strains like 569 B. Molecular genetic analysis of classical biotype *V. cholerae* strains that caused cholera outbreaks in 1942 in Russia showed that these strains contain the gene *hapA,* demonstrated by PCR, but produce no soluble HAP [30]. It would be interesting to study the presence and role of the 59-kDa serine protease in such classical *V. cholerae* O1 strains.

The genus *Vibrio* consists of many pathogenic species that include *V. cholerae*, *V. parahemolyticus*, *V. vulnificus, V. mimicus* and *V. fluialis* [31]. In addition to toxins and hemolysin produced by vibrios, protease is also recognized as one of the pathogenic factors in some *Vibrio* species [3]. The proteases in vibrios are divided into two main groups, the zinc metalloproteases and the serine proteases. *V. cholerae* and *V. vulnificus* secrete proteases belonging to the thermolysin family of metalloproteases which have a zinc ion and are immunologically cross-reactive with each other [3]. There are no studies on the role of serine protease in *V. cholerae*. Existence of the thermolysin family of zinc metalloproteases has not been recognized in *V. parahemolyticus*, although the production of other kinds of proteases including serine proteases have been reported [32]. A 50-kDa serine protease designated as VPPI (*Vibrio parahemolyticus* protease I) was purified from the culture supernatant of a clinical strain of *Vibrio parahemolyticus* [32]. VPPI activity was inhibited by EDTA, EGTA and serine protease inhibitors, but not when EDTA was incubated in the presence of CaCl_2_ suggesting that it is a calcium- dependent serine protease [32]. The N-terminal amino acid sequence of VPPI was quite similar to that of the *Vibrio metschnikovii* protease. It was also demonstrated that VPPI or its related proteases is widely distributed in not only *V. parahemolyticus* but also *V. alginolyticus. V. parahaemolyticus* protease possesses various toxic activities, including the collagenolytic, cytotoxic, hemolytic and edema-forming activity [33]. *V. vulnificus* also secretes a 59-kDa serine protease, which is the free form, while the 69-kDa protein may be a complex form associated non-covalently with small peptide(s) [34]. The proteolytic activity of the final preparation was almost completely abolished by treatment with 5 mM PMSF, a well-known inhibitor of serine proteases. By contrast, tetraethylenepentamine, a specific inhibitor of metalloproteases including VVP (*Vibrio vulnificus* protease), showed no inhibitory effect on the proteolytic activity. *Vibrio vulnificus* strain NCIMB 2137, in which *vvp* has been deleted, secretes a serine protease, VVA0302. The serine protease, VVA0302, is an orthologue of an extracellular protease produced by *V. parahaemolyticus*. *V. vulnificus* serine protease may be a virulence factor in vibriosis, which is characterized by external and internal hemorrhages affecting the major organs [35], or human wound infection with necrotic tissue damage [36].

Our results show the presence of several proteases in *V. cholerae*, such as HAP, PrtV, 59-kDa serine protease and other novel proteases. The serine protease from a Δ*hapA*Δ*prtV V. cholerae* O1 strain induced hemorrhagic response in rabbit ileal loop. The strains used for rabbit ileal loop experiments were grown in tryptic soy broth under conditions which are not optimal for CT production (Results not shown). Although Young and Broadbent [16] had earlier reported that strain 569B secretes a protease that is inhibited by PMSF, our study may be the first to demonstrate the presence of a novel 59-kDa serine protease in *V. cholerae* and its role in hemorrhagic response in RIL model. Studies have shown wide variation in extracellular protease production among different strains of *V. cholerae* [16]. Among *V. cholerae* El Tor strains, there was a 100-fold variation in protease production and the two classical strains tested differed in protease production by a factor of 80 [16]. Further studies are in progress to characterize the expression levels of this 59-kDa serine protease in *V. cholerae* strains of both El Tor and classical biotypes.
